# A Carrier for Non-Covalent Delivery of Functional Beta-Galactosidase and Antibodies against Amyloid Plaques and IgM to the Brain

**DOI:** 10.1371/journal.pone.0028881

**Published:** 2011-12-21

**Authors:** Gobinda Sarkar, Geoffry L. Curran, Eric Mahlum, Teresa Decklever, Thomas M. Wengenack, Anthony Blahnik, Bridget Hoesley, Val J. Lowe, Joseph F. Poduslo, Robert B. Jenkins

**Affiliations:** 1 Department of Experimental Pathology, Mayo Clinic, Rochester, Minnesota, United States of America; 2 Department of Orthopedics, Mayo Clinic, Rochester, Minnesota, United States of America; 3 Department of Neurology, Mayo Clinic, Rochester, Minnesota, United States of America; 4 Department of Radiology, Mayo Clinic, Rochester, Minnesota, United States of America; 5 Tissue and Cell Molecular Analysis Laboratory, Mayo Clinic, Rochester, Minnesota, United States of America; Massachusetts General Hospital and Harvard Medical School, United States of America

## Abstract

**Background:**

Therapeutic intervention of numerous brain-associated disorders currently remains unrealized due to serious limitations imposed by the blood-brain-barrier (BBB). The BBB generally allows transport of small molecules, typically <600 daltons with high octanol/water partition coefficients, but denies passage to most larger molecules. However, some receptors present on the BBB allow passage of cognate proteins to the brain. Utilizing such receptor-ligand systems, several investigators have developed methods for delivering proteins to the brain, a critical requirement of which involves covalent linking of the target protein to a carrier entity. Such covalent modifications involve extensive preparative and post-preparative chemistry that poses daunting limitations in the context of delivery to any organ. Here, we report creation of a 36-amino acid peptide transporter, which can transport a protein to the brain after routine intravenous injection of the transporter-protein mixture. No covalent linkage of the protein with the transporter is necessary.

**Approach:**

A peptide transporter comprising sixteen lysine residues and 20 amino acids corresponding to the LDLR-binding domain of apolipoprotein E (ApoE) was synthesized. Transport of beta-galactosidase, IgG, IgM, and antibodies against amyloid plques to the brain upon iv injection of the protein-transporter mixture was evaluated through staining for enzyme activity or micro single photon emission tomography (micro-SPECT) or immunostaining. Effect of the transporter on the integrity of the BBB was also investigated.

**Principal Findings:**

The transporter enabled delivery to the mouse brain of functional beta-galactosidase, human IgG and IgM, and two antibodies that labeled brain-associated amyloid beta plaques in a mouse model of Alzheimer's disease.

**Significance:**

The results suggest the transporter is able to transport most or all proteins to the brain without the need for chemically linking the transporter to a protein. Thus, the approach offers an avenue for rapid clinical evaluation of numerous candidate drugs against neurological diseases including cancer. (299 words).

## Introduction

Numerous potential drug candidates for treating brain-associated disorders involving mood, behavior, addiction, aging, infection, cancer and neurodegenerative disease exist but therapeutic use of these candidate drugs currently remains unrealized due to serious impediment imposed by the blood-brain-barrier (BBB) [1]–[9]. The existence of the BBB was reported over a century ago [10]. Transport of small molecules, typically <600 daltons is generally allowed by the BBB, whereas passage of larger molecules is usually restricted. Several receptors present on the BBB are known to allow passage of cognate protein ligands to the brain [11]–[13]. Such receptor-ligand systems on the BBB have been reportedly utilized to develop strategies for delivering target proteins in the brain. All these approaches, however, rely on covalent linking of a carrier peptide resembling the receptor-binding domain of a ligand [14]–[16] or an antibody resembling the ligand [17], [18], to the target protein of interest. Other approaches utilizing different peptides or proteins as transporters also require covalent linking of a protein ‘load’ to the transporter for delivery across the BBB [19]–[21].

Our previous efforts at developing avenues for increased delivery across the BBB also depended upon covalent linking of a protein to polyamines [22], [23], or through synthetic insertions of asparagyl/glutamyl-4-amino-butane [24]. There are considerable technical and other challenges associated with covalent linking of a protein to a carrier molecule in the context of delivery across the BBB, which, conceivably, has limited translational applications of the existing methods. Consequently, our objective was to develop a method abolishing the requirement for covalent modification of a target protein to be delivered across the BBB. We reasoned that to achieve such an objective requires a transporter that fulfills at least two criteria: it should bind strongly to a target protein in a non-covalent manner *and* it should be able to ‘piggyback’ the bound protein across the BBB.

We have previously shown that a stretch of sixteen lysine residues (K16) can non-covalently and strongly bind to proteins. When the K16 stretch was linked with the signal peptide sequence of Kaposi's Fibroblast Growth factor, the resulting peptide delivered the bound proteins into cells [25]. Thus, the use of K16 would fulfill our first key requirement. To meet the second requirement, we elected to use the low-density lipoprotein receptor (LDLR)-binding 20-amino acid segment of apolipoprotein E (ApoE peptide) comprising amino acids 151–170 (Swiss-Prot # P02649). When covalently linked, this peptide can deliver glucocerebrosidase to the brain through LDLR-mediated transcytosis [16]. Subsequently, a bi-partite peptide was synthesized comprising the ApoE peptide linked with sixteen lysine residues (henceforth K16ApoE), which was evaluated for its potential for non-covalent transport of proteins across the BBB. We demonstrate successful K16ApoE-mediated non-covalent delivery of three different proteins (beta-galactosidase, IgG and IgM) across the BBB. To our knowledge this is the first report demonstrating successful delivery of various proteins across the BBB that does not involve chemically linking the proteins with a carrier entity.

## Materials and Methods

### Materials

Mice were maintained and used following Institutional Animal Care and Use Committee (IACUC)-approved protocol # A3291-01. All mice used (B6SJLF1) were female and were purchased from the Jackson Laboratories. Bacterial beta-galactosidase was purchased from Calbiochem (Catalog # 345788). Human IgG and IgM were purchased from Sigma (Product Numbers I 4506 and I8260 respectively). The 4G8 monclonal antibody (cat# SIG-39220) was purchased from Covance (Emeryville, CA). LDL receptor antibody was from abcam (Cat # ab30532).

All peptides were synthesized at the Mayo Proteomic Core Facility. The transporter peptide, K16ApoE, had NH2 at both ends. The transporter peptide has the following amino acid sequence (in single-letter code): KKKK KKKK KKKK KKKK LRVR LASH LRKL RKRL LRDA.

### Preparation of peptide-protein complex for delivery in the brain

Required amount of the peptide and protein were mixed in a final volume of 300 uL PBS (phosphate buffered saline), and incubated at room temperature for 60 min. The mixture was vortexed for a few seconds, every fifteen minutes during the incubation period. The mixture was somewhat turbid at high peptide and protein concentration.

The mixture was injected intravenously as a bolus into the lumen of the femoral vein. (K16ApoE-mediated delivery of proteins to the brain through tail-vein iv injection has not been explored at this time). This was accomplished using a heat pulled PE50 catheter. At the conclusion of the experiment, the mouse was euthanized with a lethal dose of sodium pentobarbital. Each mouse was perfused with 10 ml PBS. This perfusion was accomplished through the standard trans cardial method [26]. The brain was removed from the skull and positioned to make an initial coronal slice at −2.0 mm bregma. Subsequently, 25 um coronal sections were cut on a cryostat and placed on charged slides for staining of beta-galactosidase activity.

### Staining for beta-galactosidase enzyme activity

Evaluation of beta-galactosidase enzymatic activity was accomplished by an initial 15 min fixation of the brain sections in 0.25% glutaraldehyde. The slides were washed with 3 changes of PBS for 5 minutes each and then rinsed in distilled water for 5 minutes. The brain sections were incubated in X-Gal (0.2%) working solution, pH 7.38, for 18 hours (overnight) at 37° C in covered containers. Following this incubation the sections were dehydrated and coverslips were applied [21], [27].

### Imaging by micro single photon emission computed tomography (microSPECT)

Micro SPECT/CT experiments were conducted on a Gamma Medica X SPECT System (GE Healthcare). Human IgG (Sigma) and IgM (Sigma) were labeled to a high specific activity using the Chloramine-T method [23]. 80 ug (corresponding to ∼420 uCi of IgG and ∼397 uCi of IgM) of each immunoglobulin (corresponds to 0.53 nanomole of IgG and 0.13 nanomole of IgM, based on molecular weights of 150 Kd and 600 Kd for IgG and IgM, respectively) was mixed with 70-fold molar excess of K16ApoE for 1 h at room temperature and was administered in each mouse through the use of a catheter in the femoral vein. Immediately subsequent to the intravenous bolus injection, the mice were imaged every hour for a total of 6 hours [28]. At the completion of the 6 h time point, each mouse was euthanized and the systemic blood supply was transcardially perfused with 10 ml phosphate buffered saline, and imaged after 30 min. Results are from 6 mice in each group.

### Immunostaining of 4G8 antibody (and other antibodies) delivered in mice brain with and without K16ApoE

Following iv injection of 4G8 IgG (Covance Research Products, Berkeley, CA), alone or with K16ApoE, mice were overdosed with sodium pentobarbital (200 mg/kg, ip) and perfused transcardially with PBS followed by neutral-buffered, 10% formalin. The brains were removed and flash frozen. Twenty-five micron sections were cut on a cryostat and thaw-mounted on gelatine coated slides. One set of slides from each mouse was reacted with biotinylated secondary anti-mouse IgG antibody, ABC immunoperoxidase (Vector M.O.M. Peroxidase Kit, PK-2200, Vector Laboratories, Burlingame, CA), and Vector VIP peroxidase substrate (SK-4600, Vector Laboratories, Burlingame, CA) to test for the presence of the 4G8 IgG in the brain. Briefly, sections were washed with tris-buffered saline, pH 7.5 (TBS). Endogenous peroxidase activity in the sections was quenched by reacting with 0.5% H_2_O_2_ in TBS for 30 min. The sections were blocked with M.O.M blocking solution in TBS for 60 min. The 4G8 IgG antibody was then visualized using standard immunoperoxidase techniques with 1 h incubation times for the biotinylated secondary antibody and ABC solutions [29].

Two sets of adjacent sections were processed histologically to detect amyloid deposits and plaques using two standard methods. One set of sections underwent immunohistochemistry for amyloid using the same 4G8 anti-APP monoclonal mouse antibody (Covance Research Products, Berkeley, CA) in vitro to verify the binding of the monoclonal IgG to amyloid plaques. Briefly, sections were washed with TBS. Endogenous peroxidase activity in the sections was quenched by reacting with 0.5% H_2_O_2_ in TBS for 30 min. The sections were blocked with M.O.M. blocking solution in TBS for 60 min. The sections were incubated with the 4G8 anti-APP primary antibody at a dilution of 1∶1000 in 0.1% BSA/0.3% Triton-X/PBS overnight at 4°C. The primary antibody was then visualized using an ABC immunoperoxidase kit (Vector M.O.M. Peroxidase Kit, PK-2200) and Vector DAB (SK-4100) as the substrate according to the instructions (Vector Laboratories, Burlingame, CA). Another set of adjacent sections was stained with fresh, filtered, aqueous 1% thioflavine S to stain the amyloid plaques using a standard protocol [29]. The thioflavine S positive amyloid plaques were visualized with epifluorescence microscopy using filters for fluorescein isothiocyanate (excitation = 488 nm; emission = 520 nm).

## Results

### The peptide transporter can non-covalently deliver functional beta-galactosidase in the brain

**Figure 1:**
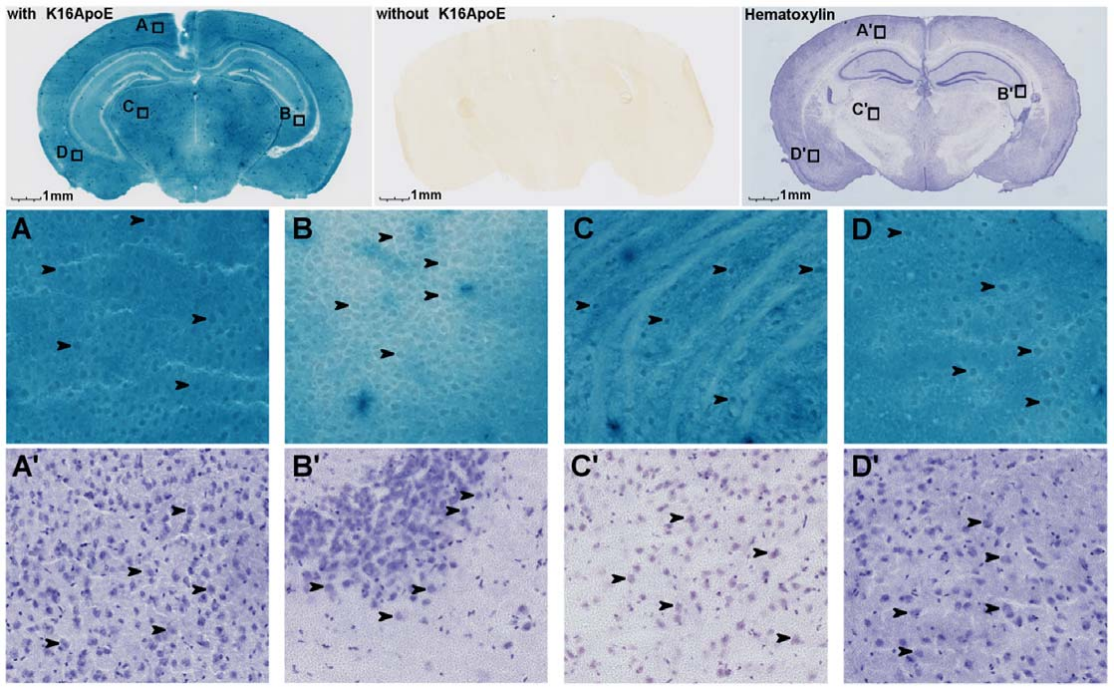
Delivery of beta-galactosidase in mouse brain through K16ApoE and visualization of cells stained for beta-galactosidase activity in different areas in the brain following delivery of the enzyme. Beta-galactosidase (one nanomole) was injected in mice mixed with K16ApoE (70 nanomoles), tissue slices made six hours after injection were stained for enzyme activity (top left). Middle image, top row –an image of a brain slice obtained after delivering beta-galactosidase without K16ApoE. Third image, top row – image of a brain slice stained with hematoxylin. Also, high magnification (20×) images from four regions of the beta-galactosidase-stained section (A,B, C and D) and hematoxylin-stained comparable regions (A′, B′, C′ and D′) are shown. A,A′ - retrosplenial agranular cortex; B,B′ - CA3 field, hippocampus; C,C′ - posterior thalamic nuclear group; D, D′ - basolateral amygdaloid nuclei, posterior. Arrows indicate some of the cells in the respective regions.

The potential of K16ApoE for delivering a protein across the BBB was first evaluated by intravenous (IV) injection of the enzyme beta-galactosidase into mice with or without prior mixing with K16ApoE (at a protein to peptide molar ratio of 1∶70, based on preliminary experiments. A wide range of protein to peptide molar ratios up to 1∶100 [higher molar ratio not tested] produced very satisfactory results). Intense beta-galactosidase activity was observed in the mouse brain when the enzyme-peptide mix was injected intravenously and brain slices were prepared for enzyme activity staining 6 h after injection,{data presented in Figure S1 demonstrate that 6 h of time is sufficient for visual evaluation of intracerebral beta-galactosidase activity after K16ApoE-mediated iv delivery in brain}whereas no activity was seen when the enzyme was injected alone (Figure 1). High-magnification scan of several regions of brain-sections stained for K16ApoE-delivered beta-galactosidase activity show that the enzyme was delivered in nearly every area of the brain, and appeared to have stained all cells when compared to cells stained with hematoxylin (compare sections A and A′, C and C′, and D and D′; Figure 1). However, pyramidal cells in the hippocampus (compare sections B and B′) appear to show much weaker staining for beta-galactosidase activity compared to cells in other areas of the brain. Whether the weak beta-galactosidase activity staining in the pyramidal cells is due to low expression of LDLR in the hippocampus remains to be determined. It is also possible that the peptide carrier utilizes multiple related receptors for transcytosis, most or all of which is expressed in most cells in the brain but cells in the hippocampus may not express all those receptors leading to reduced uptake of a protein ‘load’. Further, it is possible that deficient staining in the hippocampus reflects enhanced leakage of X-gal and/or 5,5′-dibromo-4,4′-dichloro-indigo (the chromophore generated upon beta-galactosidase action on X-gal) from the pyramidal cells in the hippocampus as compared with cells in other brain regions. Delivery of proteins across the BBB whose functional identification does not depend upon small molecule substrate or product might help resolve this issue. These data, however, strongly show that the peptide transporter can transport enzymatically functional beta-galactosidase across the BBB and into the brain in a simple ‘mix-and-inject’ manner.

### Delivery of beta-galactosidase to the brain by the transporter appears to be mediated by the LDLR

**Figure 2:**
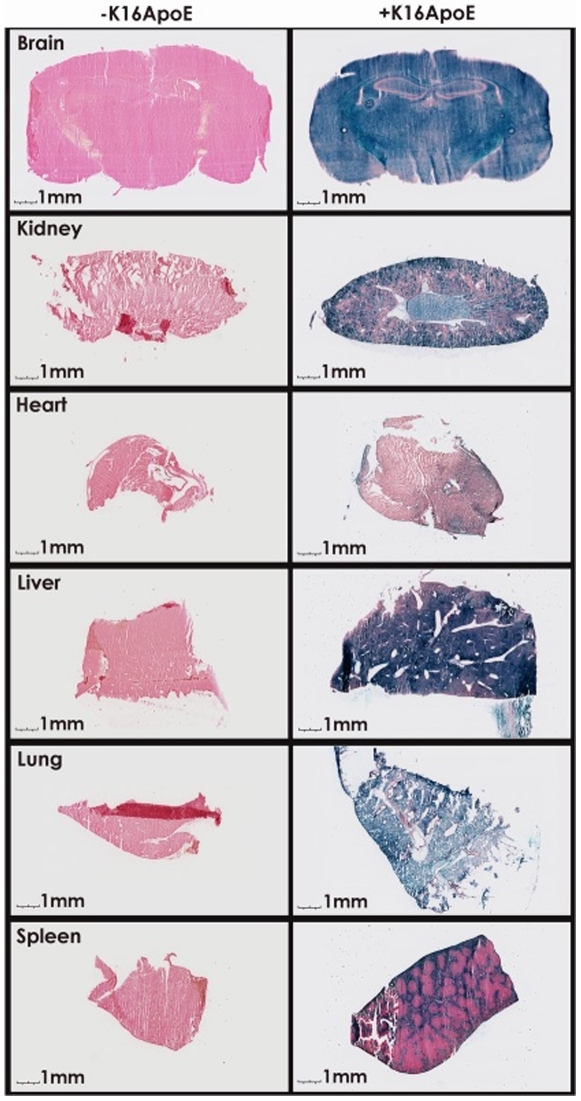
K16ApoE-mediated delivery of beta-galactosidase in various organs. Slides of 25 micron thickness were made with tissues from different organs and stained for beta-galactosidase activity after delivery. Left panel – no K16ApoE; right panel – with K16ApoE.

To assess if the delivery of beta-galactosidase in the brain via K16ApoE is LDLR-mediated, we evaluated beta-galactosidase staining in liver, kidney, heart, spleen and lung after intravenous injection of the enzyme mixed with K16ApoE (Figure 2). The most intense staining was observed in liver followed by staining in lung, kidney spleen and heart. The pattern of cellular uptake of beta-galactosidase in these organs by K16ApoE is strongly correlated with reported LDLR expression patterns in various tissues (high in liver and brain, low in other organs) [30]–[32], [16], suggesting that delivery of beta-galactosidase via K16ApoE is LDLR-mediated. Use of LDLR knockout animals should provide important information in deciphering the mechanism underlying K16ApoE-mediated transport of proteins across the BBB. It also remains to be seen if K16ApoE-mediated delivery of proteins across the BBB takes place exclusively through the LDLR; use of LDLR-knockout animals should help resolve the issue.

### Non-covalent delivery of IgG and IgM by the transporter across the BBB

**Figure 3:**
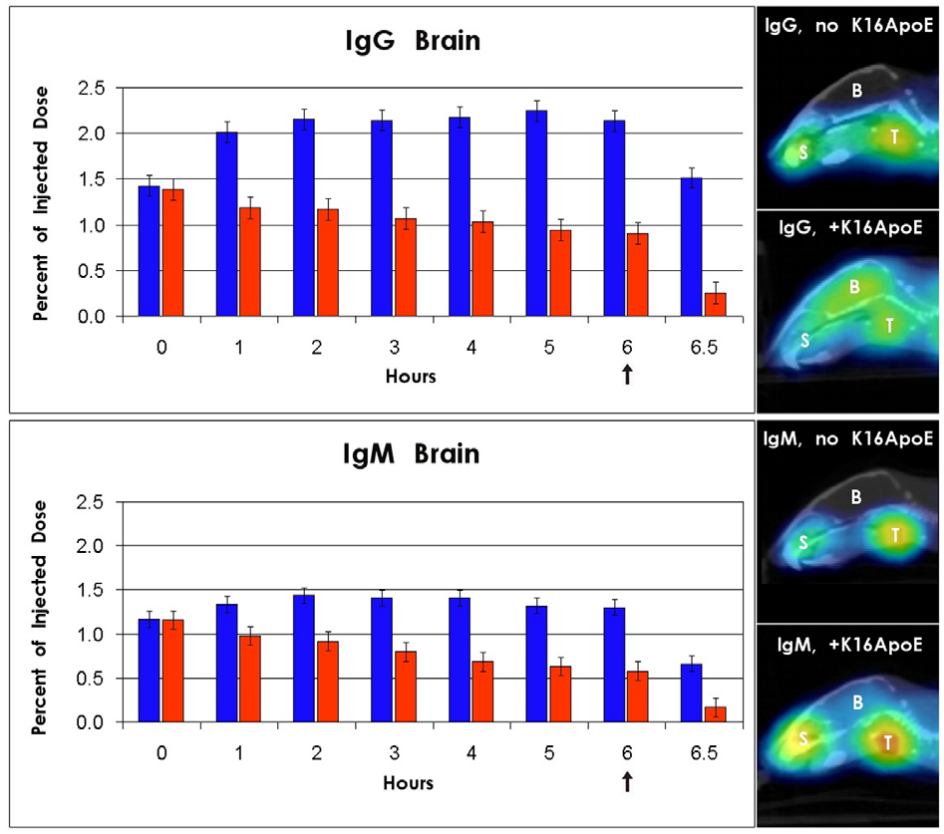
Evaluation of K16ApoE-mediated delivery of IgG (A) and IgM (B) in brain by microSPECT. Imaging was done at 1 h interval up to six hours, after which cardiac perfusion was performed, and final imaging carried out 30 min after perfusion. Blue bars – delivery with K16ApoE; red bars – delivery without K16ApoE. The upward arrows at time 6 h indicate time when cardiac perfusion was performed. Right panel – microSPECT images of mice head after perfusion after delivery of radiolabeled IgG and IgM. B – brain; S – salivary gland; T – thyroid.

Since many potentially therapeutic proteins against brain-associated disorders are immunoglobulins, we evaluated delivery of normal human serum IgG and IgM with K16ApoE in mouse brain by micro-single photon emission computed tomography (microSPECT) imaging (Figure 3). For this, radioiodinated human IgG and human IgM were mixed with and without K16ApoE, and injected intravenously. Anesthetized animals were then subjected to imaging at 1 h intervals for 6 h at which time cardiac perfusion was done. Final imaging was performed at 30 min post-perfusion. Several observations can be made from these experiments: First, it appears that peak brain delivery of both IgG (Figure 3A) and IgM (Figure 3B) occurs at 2 h after injection of the peptide-protein mix, and then remains relatively stable up to 6 h (maximum time point tested). Second, cardiac perfusion appears to remove most of the radioactive protein in the vasculature, concomitantly increasing the *difference* between apparent brain-uptake of the protein with and without the transporter peptide (4.89-fold for IgG (p-value<0.0001) and 3.94 fold for IgM (p-value 0.0009, see Table 1). This is probably a lower estimate of the efficiency of delivery across the BBB by the transporter, because complete removal of the vasculature-bound labeled IgG and IgM (the numerical value which is the denominator in the estimation) by cardiac perfusion was not achieved. That the efficiency of delivery is high is supported by our beta-galactosidase-based experiments since no animal receiving beta-galactosidase injection without the transporter showed any visually detectable intra-cerebral enzyme activity. Finally, we note that uptake of the radiolabeled IgG and IgM into kidney, liver/spleen and heart did not appear to depend on whether the proteins were delivered with or without the transporter (Figure S2). Cardiac perfusion also did not change the accumulation of radioactivity in these organs, implying that accumulation of proteins in these organs does not depend upon crossing of a biological barrier harboring LDLR.

**Table 1:** Evaluation of peptide transporter-mediated uptake of IgG and IgM*.

Protein delivered	Time (hr)	Fold-Change**	P-Value
IgG	0	0.96	0.3523
	1	1.53	<0.0001
	2	1.7	<0.0001
	3	1.79	<0.0001
	4	1.91	<0.0001
	5	2.08	<0.0001
	6	1.83	<0.0001
	6.5	4.89	<0.0001
IgM	0	1.01	0.8617
	1	1.37	0.0004
	2	1.56	0.0013
	3	1.76	0.0005
	4	2.07	0.0017
	5	2.09	0.0021
	6	2.24	0.0008
	6.5	3.94	0.0009

*Mice were injected with the radiolabeled immunoglobulin with or without prior mixing with the peptide transporter. MicroSPECT imaging was done from 0 to 6 h without perfusion. Cardiac perfusion was carried out at 6 h post-injection and final imaging was done 30 min later. Six mice were used in each group.

**Fold-Change refers to uptake with the transporter divided by uptake without the transporter.

### IgG delivered by the transporter across the BBB can recognize a specific ligand in the brain

**Figure 4:**
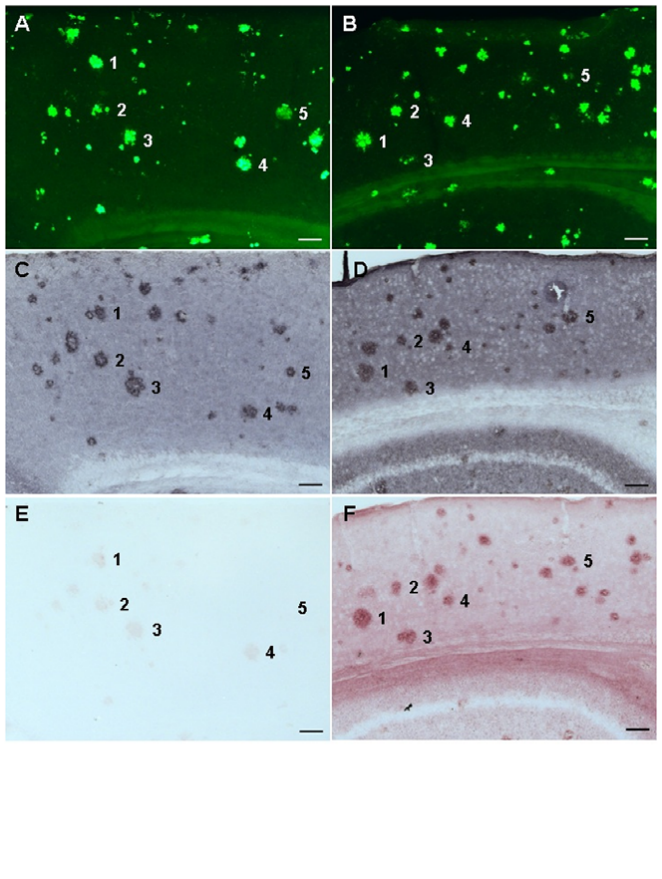
Labeling of amyloid plaques with a plaque-specific antibody (4G8) delivered via K16ApoE in brains of mice models of Alzheimer's disease (AD). Two mice with AD were used: one represented by A, C and E are adjacent brain sections from one mouse, while adjacent sections from the other mouse are represented by B, D and F. A, B – thioflavine S staining; C, D – immunostaining to identify plaques using the 4G8 as the primary antibody, and an anti-mouse antibody as the secondary antibody; E, F – immunostaining using the secondary antibody only. The 4G8 IgG was injected in the first mouse (left panel) without K16ApoE, while the second mouse (right panel) received injection of the IgG mixed with K16ApoE. Numbers 1–5 represents approximately corresponding plaques. Scale bar – 100 micrometer.

The radiolabeled immunoglobulins delivered into the brain through K16ApoE could represent trapped molecules in the endothelial cells at the BBB and may not reflect entry of the delivered proteins into the brain parenchyma. Furthermore, assessment of K16ApoE-delivered beta-galactosidase activity in the brain was independent of binding of the enzyme with any intra-cerebral molecular entity. For therapeutic function, most proteins transported across the BBB must be delivered to the brain parenchyma and be able to recognize and bind with a cognate target molecule. To test binding specificity of the delivered protein, we mixed an antibody against amyloid beta peptide (4G8) with K16ApoE and injected the mixture intravenously into APP/PS1 mice (a model for Alzheimer's disease). This antibody is known to recognize amyloid beta plaques [33]. Results presented in Figure 4 and Figure S3 indicate that the K16-ApoE-delivered antibody labeled the amyloid plaques in the brain of APP/PS1 mice in a manner nearly identical to parallel sections stained by the plaque-specific dye ThioflavinS or by standard immunohistochemistry with the same antibody. The results imply that the 4G8 antibody was specifically targeted to the amyloid plaques via K16ApoE-mediated delivery. Similar results were also obtained with another antibody (IgG4.1) known to label amyloid plaques (Figure S4) [26]. Collectively, these results show that an antibody delivered in the brain via K16ApoE can be targeted to neurons. In a separate experiment, the 4G8 IgG delivered via K16ApoE in a normal mouse brain was negative for any staining (Figure S5). In a related experiment, a monoclonal antibody L227, not specific to the amyloid plaques [34], did not appear to label any amyloid plaques when delivered to the brain of an AD-mouse via K16ApoE (Figure S6).

### The peptide transporter K16ApoE does not impair the BBB

**Figure 5:**
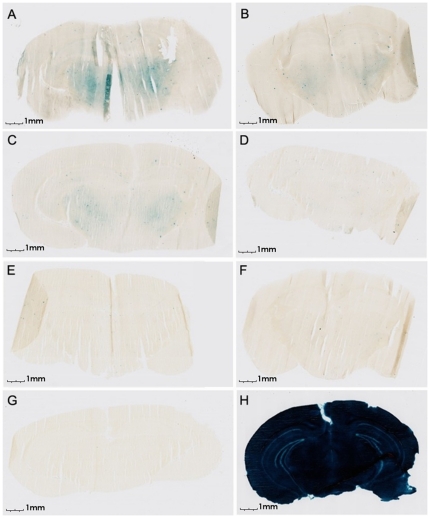
Evaluation of the integrity of the BBB after injection of K16ApoE. Separate mouse (represented by A through G) all received an injection of K16ApoE, followed by injection of beta-galactosidase at indicated times. Brain slices were made 6 h after beta-galactosidase injection and proceeded with staining for activity of the enzyme. A – Beta-galactosidase was injected 1 min after K16ApoE injection. B – Beta-galactosidase was injected 5 min after K16ApoE injection. C – Beta-galactosidase was injected 10 min after K16ApoE injection. D – Beta-galactosidase was injected 30 min after K16ApoE injection. E – Beta-galactosidase was injected 1 h after K16ApoE injection. F – Beta-galactosidase was injected 2 h after K16ApoE injection. G – Beta-galactosidase was injected 4 h after K16ApoE injection. H- positive control (beta-galactosidase mixed with K16ApoE was injected, brain slices were made 4 h after the injection and stained for enzyme activity.

For therapeutic and/or diagnostic use, a transporter that efficiently carries a protein in the brain should not impair the integrity of the BBB. To evaluate integrity of the BBB upon K16ApoE-mediated delivery, we first injected the peptide intravenously, then injected beta-galactosidase at different time intervals. The control mice received the enzyme mixed with the peptide. Mice were perfused and sacrificed 6 h after injection of beta-galactosidase, brain slices were prepared and subjected to beta-galactosidase staining. Results presented in Figure 5 show successively weaker beta-galactosidase activity in the brains of mice receiving beta-galactosidase at 1, 5 and 10 min after injection of K16ApoE and no visible staining thereafter. Our interpretation of these results is that most of the injected K16ApoE binds with proteins and cells in the circulation. This intravascular binding is virtually complete within 10 min, after which no free K16ApoE remains in the circulation. Free K16ApoE that remains at early time points becomes bound with beta-galactosidase and carries the enzyme in the brain. The peptide by itself and/or being bound with blood proteins/cells does not seem to affect the BBB since no beta-galactosidase enzyme activity is seen in the brain from 10 min to 6 h. An alternative explanation is that the transporter does compromise the BBB, but the damage is spontaneously repaired in a short time (within ∼10 mins). We note that animals receiving iv injection of K16ApoE alone or mixed with beta-galactosidase, IgG or IgM showed no visually detectable behavioral or physiological impairment for up to 24 h (longest time observed). It remains to be seen if K16ApoE compromises the integrity of the BBB in the context of passage of small molecules.

## Discussion

We have demonstrated the ability of a synthetic peptide, K16ApoE, as a transporter of target proteins in the brain employing a simple ‘mix-and-inject’ approach. The most notable aspect of the transporter, unlike current methods, is that it does not need to be chemically linked to a protein ‘load’ to be transported across the BBB. Thus, K16ApoE may be regarded as a universal protein delivery agent for the brain. Transport of proteins to the brain by K16ApoE was evaluated by employing three different strategies (enzyme activity, radiologic imaging and immunostaining), and by delivering three different proteins of diverse molecular weight. As far as we know, this is the first report of a delivery reagent, targeting uptake of a protein by the brain that does not require any chemical linking between the transporter and the protein. We have delivered functional beta-galactosidase to the brains of >100 mice with this peptide transporter, and successful delivery have been observed in every instance.

For clinical applications, a protein that has been transported to the brain must be able to recognize and bind with an intracranial target molecule and remain biologically functional. By delivering anti-amyloid antibodies in the brains of AD mice through K16ApoE, and observing that the delivered antibodies label the amyloid plaques in a manner almost indistinguishable to identifying the plaques through standard immunohistochemistry coupled with the demonstration that the delivered molecules retains biological activity (results of the beta-galactosidase based experiments) raises the possibility that K16ApoE has potential for clinical applications. It is intriguing to note that despite availability of methods for delivering proteins across the BBB for nearly two decades, successful translational application of these methods, to date, remains challenging. The limitation imposed by the requirement of chemically conjugating a target protein to a transporter entity may underlie such a scenario. Since our peptide transporter specifically abolishes the requirement of chemical linkage between a transporter and a protein load, we believe our peptide transporter will allow rapid pre-clinical evaluation of numerous potential protein-drugs currently in the pipeline.

We have not, so far, specifically addressed quantifying the fraction of injected dose of a protein that ultimately is transported to the brain parenchyma through K16ApoE. We speculate that to achieve such quantification, the contribution of the vessel-bound target protein that remains even after perfusion needs to be completely eliminated. Results presented in Figure 3 indicate that ∼1.5% of the immunoglobulins injected entered the brain. Further experiments are in progress along this line. Whether delivery of this magnitude will have any clinical significance remains to be seen, however, since we observe intense beta-galactosidase staining in virtually all areas of the brain, and that most or all beta-amyloid plaques in the brain become labeled with antibodies delivered in the AD mice through the transporter, we remain hopeful that the transporter will have reasonable clinical relevance.

In addition to providing evidence for a simple reagent as transporter of proteins across the BBB, our approach also illustrates a general strategy to develop similar reagents based upon ligand-receptor systems on the BBB. Conceivably, varying the length and composition of the polybasic amino acid moiety coupled with varying the length of the ApoE moiety might yield even better transporter than K16ApoE. Discovery of BBB-specific ligand-receptor system, if any, that functions like the ApoE-LDLR, should provide avenues to deliver therapeutic proteins in the brain without affecting other organs. It remains to be seen if proteins delivered in this manner will have therapeutic effects against brain-associated disorders.

## Supporting Information

Figure S1 **Time course for beta-galactosidase delivery in mouse brain with K16ApoE.** In each animal, one nanomole of beta-galactosidase was mixed with 70 nanomoles of K16ApoE and injected intravenously. Brain slices were prepared for staining for beta-galactosidase activity at indicated time points. A- Beta-galactosidase, no K16ApoE, 6 h. B- Beta-galactosidase+ K16ApoE, 1 h. C- Beta-galactosidase+ K16ApoE, 2 h. D- Beta-galactosidase+ K16ApoE, 6 h. E- Beta-galactosidase+ K16ApoE, 10 h.
(TIFF)

Figure S2 **microSPECT images of accumulation of ^125^IgG and ^125^IgM in various organs.** In such experiments, spleen and liver could not be adequately separated. Consequently, these two organs were collected as one entity. Imaging (and, therefore, counting of radioactivity) was done at 1 h interval up to 6 h, at which time cardiac perfusion was done. A final imaging was done 30 min after perfusion. Left panel –IgG; right panel –IgM. Blue bars – with K16ApoE; red bars – without K16ApoE. Arrows indicate time at which cardiac perfusion was done.
(TIFF)

Figure S3 **Labeling of amyloid plaques with 4G8 antibody delivered with and without K16ApoE in mice models of Alzheimer's disease.** Two mice with AD were used: A, C and E represent adjacent brain sections from one mouse, whereas B, D and F represent adjacent brain sections from another mouse. A, B – thioflavine S staining; C, D – immunostaining to identify plaques using the 4G8 as the primary antibody, and an anti-mouse antibody as the secondary antibody; E, F – immunostaining using the secondary antibody only. The 4G8 IgG was injected in the first mouse (left panel) without K16ApoE, while the second mouse (right panel) received injection of the IgG mixed with K16ApoE.
(TIFF)

Figure S4 **Labeling of amyloid plaques with IgG4.1 delivered with and without K16ApoE in mice models of Alzheimer's disease.** Two mice with AD were used: A, C and E represent adjacent brain sections from one mice, whereas B, D and F represent adjacent brain sections from the second mouse. A, B – thioflavine S staining; C, D – immunostaining to identify plaques using the IgG4.1 as the primary antibody, and an anti-mouse antibody as the secondary antibody; E, F – immunostaining using the secondary antibody only. The IgG4.1 was injected in the first mouse (left panel) without K16ApoE, while the second mouse (right panel) received injection of the IgG mixed with K16ApoE.
(TIFF)

Figure S5 **Labeling of amyloid plaques with 4G8 IgG delivered with K16ApoE in normal mouse and in a mouse model of Alzheimer's disease.** Two mice were used in the experiment: one normal mouse represented by A, C and E, and one mouse with AD represented by B, D and F. A, C and E represent adjacent brain sections from the normal mouse, whereas B, D and F represent adjacent brain sections from the AD mouse. A, B – thioflavine S staining; C, D – immunostaining to identify plaques using the 4G8 IgG as the primary antibody, and an anti-mouse antibody as the secondary antibody; E, F – immunostaining using the secondary antibody only. The 4G8 IgG was injected in the two mice mixed with K16ApoE. Numbers indicate corresponding plaques. Scale bar – 100 micrometer.
(TIFF)

Figure S6 **Evaluation of specificity of labeling of amyloid plaques with a plaque-specific antibody IgG4.1 (right panel) and a non-specific control (isotype) antibody L227 (left panel) delivered via K16ApoE in the brains of mice models of Alzheimer's disease (AD).** Two mice with AD were used: one represented by A and C, which are adjacent brain sections from one mouse, while adjacent sections from the other mouse are represented by B and D. A,B - thioflavine S staining; C,D - immunostaining using the secondary antibody only. The L227 IgG was injected in the first mouse (left panel) mixed with K16ApoE, while the second mouse (right panel) received injection of the IgG4.1 mixed with K16ApoE. Numerous plaques were labeled by IgG4.1 (panel D) but none were apparently labeled by the L227 antibody (panel C). Numbers represent approximately corresponding plaques. Scale bar – 100 micrometer.
(TIF)
