# Supplementary figures and images for: A Carrier for Non-Covalent Delivery of Functional Beta-Galactosidase and Antibodies against Amyloid Plaques and IgM to the Brain
Source: PLoS One. 2011 Dec 21;6(12):e28881. doi: 10.1371/journal.pone.0028881 (PMC3244419; doi:10.1371/journal.pone.0028881)

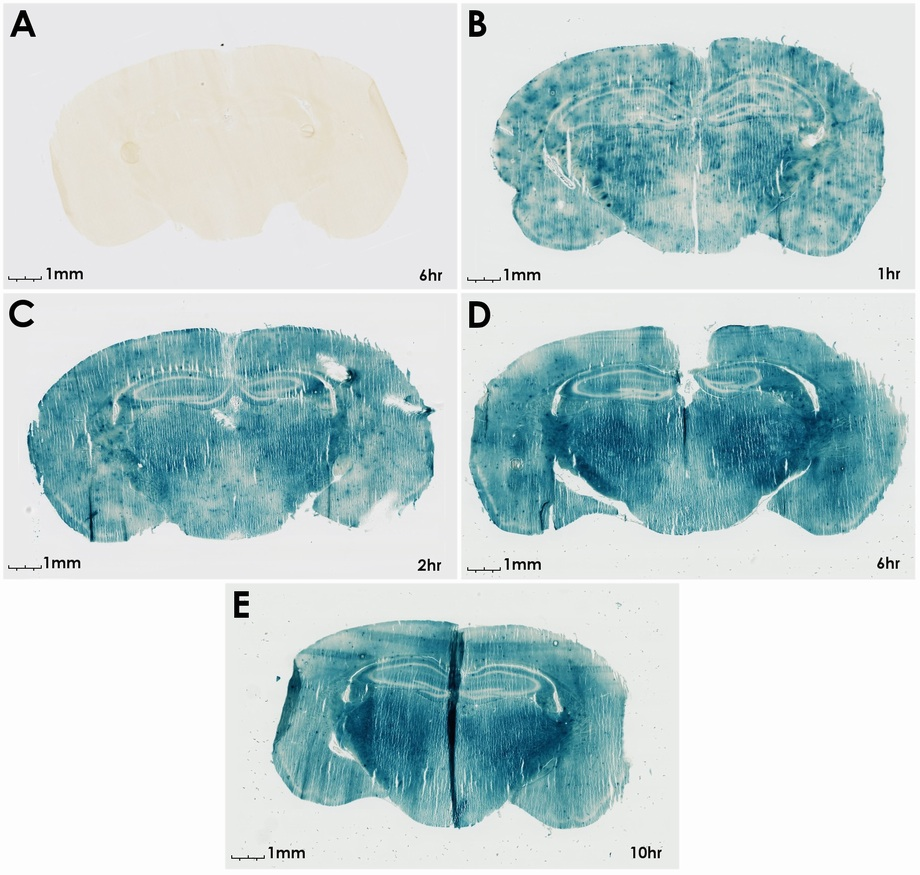

Supplement: Figure S1 — Time course for beta-galactosidase delivery in mouse brain with K16ApoE. In each animal, one nanomole of beta-galactosidase was mixed with 70 nanomoles of K16ApoE and injected intravenously. Brain slices were prepared for staining for beta-galactosidase activity at indicated time points. A- Beta-galactosidase, no K16ApoE, 6 h. B- Beta-galactosidase+ K16ApoE, 1 h. C- Beta-galactosidase+ K16ApoE, 2 h. D- Beta-galactosidase+ K16ApoE, 6 h. E- Beta-galactosidase+ K16ApoE, 10 h. (TIFF) [file pone.0028881.s001.tif]

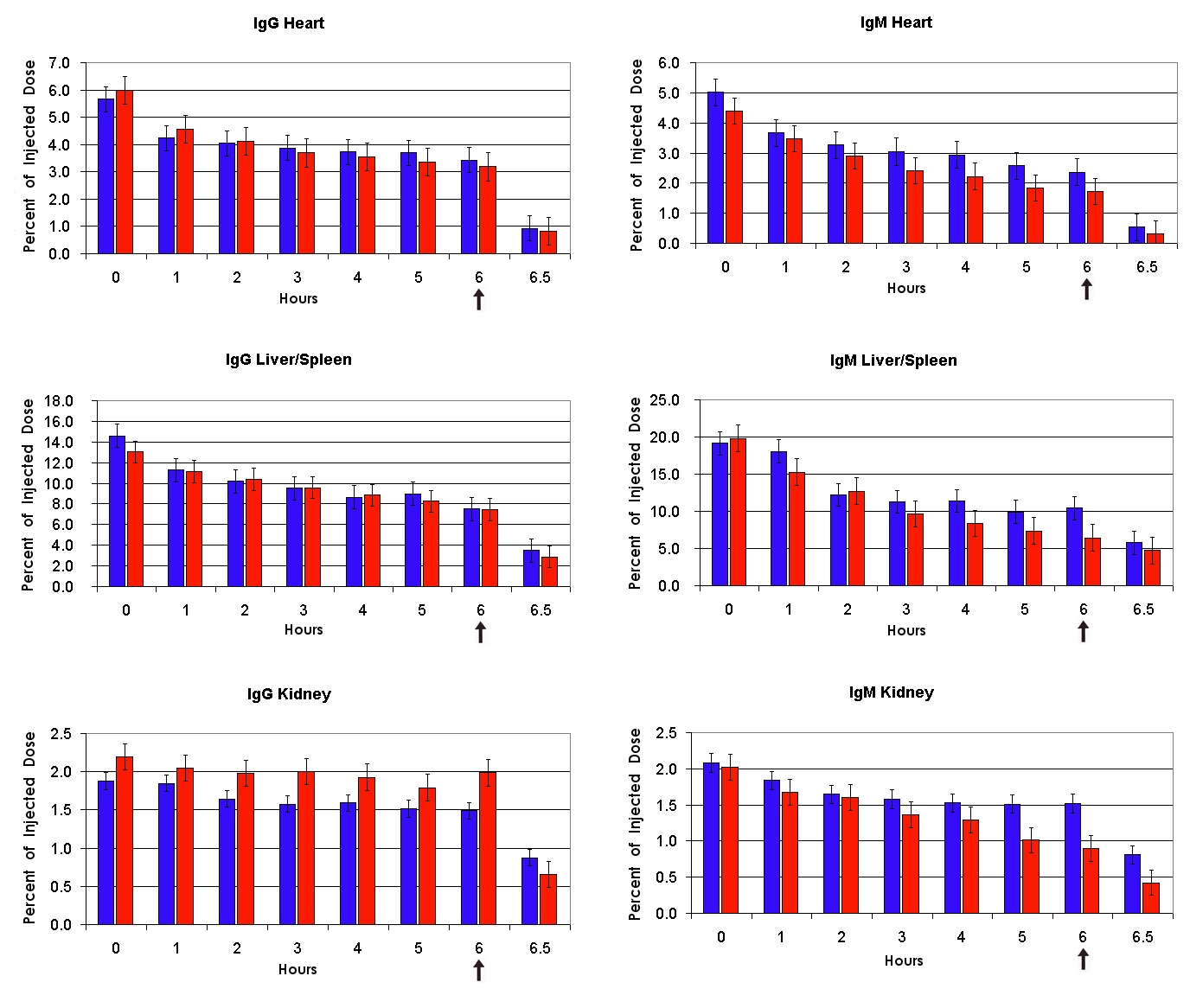

Supplement: Figure S2 — microSPECT images of accumulation of 125IgG and 125IgM in various organs. In such experiments, spleen and liver could not be adequately separated. Consequently, these two organs were collected as one entity. Imaging (and, therefore, counting of radioactivity) was done at 1 h interval up to 6 h, at which time cardiac perfusion was done. A final imaging was done 30 min after perfusion. Left panel –IgG; right panel –IgM. Blue bars – with K16ApoE; red bars – without K16ApoE. Arrows indicate time at which cardiac perfusion was done. (TIFF) [file pone.0028881.s002.tif]

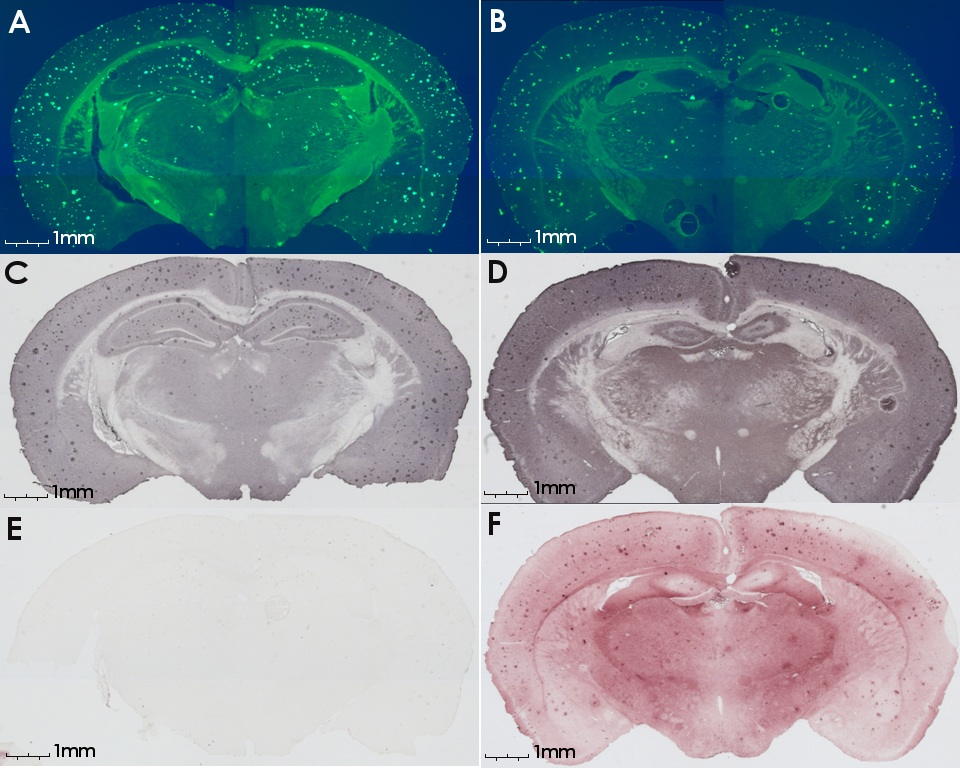

Supplement: Figure S3 — Labeling of amyloid plaques with 4G8 antibody delivered with and without K16ApoE in mice models of Alzheimer's disease. Two mice with AD were used: A, C and E represent adjacent brain sections from one mouse, whereas B, D and F represent adjacent brain sections from another mouse. A, B – thioflavine S staining; C, D – immunostaining to identify plaques using the 4G8 as the primary antibody, and an anti-mouse antibody as the secondary antibody; E, F – immunostaining using the secondary antibody only. The 4G8 IgG was injected in the first mouse (left panel) without K16ApoE, while the second mouse (right panel) received injection of the IgG mixed with K16ApoE. (TIFF) [file pone.0028881.s003.tif]

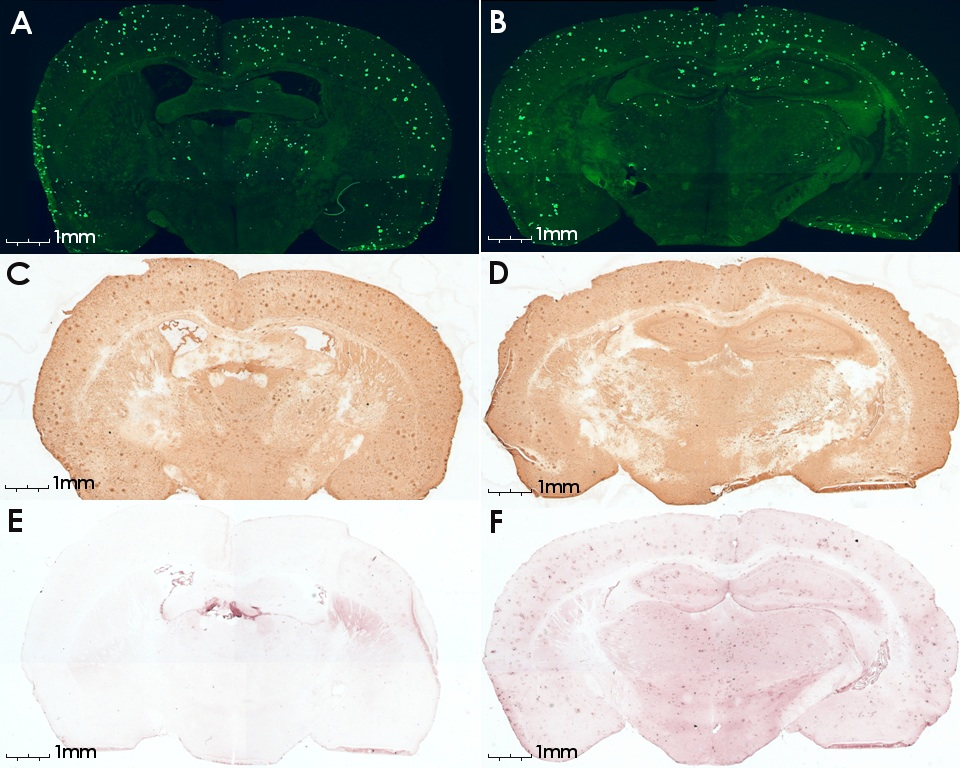

Supplement: Figure S4 — Labeling of amyloid plaques with IgG4.1 delivered with and without K16ApoE in mice models of Alzheimer's disease. Two mice with AD were used: A, C and E represent adjacent brain sections from one mice, whereas B, D and F represent adjacent brain sections from the second mouse. A, B – thioflavine S staining; C, D – immunostaining to identify plaques using the IgG4.1 as the primary antibody, and an anti-mouse antibody as the secondary antibody; E, F – immunostaining using the secondary antibody only. The IgG4.1 was injected in the first mouse (left panel) without K16ApoE, while the second mouse (right panel) received injection of the IgG mixed with K16ApoE. (TIFF) [file pone.0028881.s004.tif]

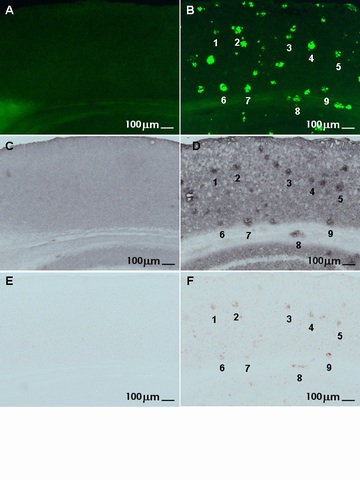

Supplement: Figure S5 — Labeling of amyloid plaques with 4G8 IgG delivered with K16ApoE in normal mouse and in a mouse model of Alzheimer's disease. Two mice were used in the experiment: one normal mouse represented by A, C and E, and one mouse with AD represented by B, D and F. A, C and E represent adjacent brain sections from the normal mouse, whereas B, D and F represent adjacent brain sections from the AD mouse. A, B – thioflavine S staining; C, D – immunostaining to identify plaques using the 4G8 IgG as the primary antibody, and an anti-mouse antibody as the secondary antibody; E, F – immunostaining using the secondary antibody only. The 4G8 IgG was injected in the two mice mixed with K16ApoE. Numbers indicate corresponding plaques. Scale bar – 100 micrometer. (TIFF) [file pone.0028881.s005.tif]

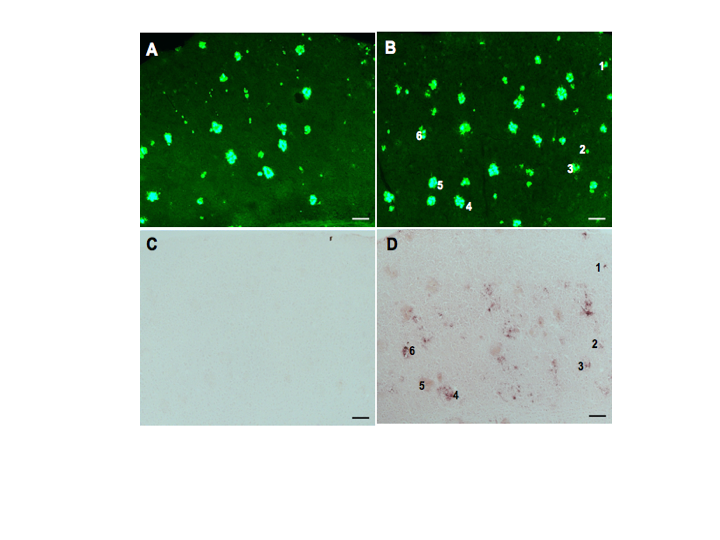

Supplement: Figure S6 — Evaluation of specificity of labeling of amyloid plaques with a plaque-specific antibody IgG4.1 (right panel) and a non-specific control (isotype) antibody L227 (left panel) delivered via K16ApoE in the brains of mice models of Alzheimer's disease (AD). Two mice with AD were used: one represented by A and C, which are adjacent brain sections from one mouse, while adjacent sections from the other mouse are represented by B and D. A,B - thioflavine S staining; C,D - immunostaining using the secondary antibody only. The L227 IgG was injected in the first mouse (left panel) mixed with K16ApoE, while the second mouse (right panel) received injection of the IgG4.1 mixed with K16ApoE. Numerous plaques were labeled by IgG4.1 (panel D) but none were apparently labeled by the L227 antibody (panel C). Numbers represent approximately corresponding plaques. Scale bar – 100 micrometer. (TIF) [file pone.0028881.s006.tif]
